# Effects of ICT use on self-regulated learning: chain intermediary effect of information retrieval and social interaction

**DOI:** 10.3389/fpsyg.2025.1637026

**Published:** 2025-09-29

**Authors:** Meiqian Wang, Shuping Zhang, Mengyuan Huang, Heping Zhang

**Affiliations:** ^1^Normal School of Vocational Techniques, Hubei University of Technology, Wuhan, China; ^2^Hubei Institute of Vocational Education Development, Hubei University of Technology, Wuhan, China

**Keywords:** ICT use, self-regulated learning, information retrieval, social interaction, intermediary effect

## Abstract

**Introduction:**

Self-regulated learning (SRL) is a crucial competency in today’s rapidly evolving digital society. Information and communication technologies (ICT) offer unprecedented opportunities to enhance SRL. However, the relationship between ICT use and SRL remains contentious. Understanding how ICT use affects teenagers’ SRL through cognitive and social pathways is essential for guiding healthy digital learning habits. This study examined the relationship between ICT use and SRL among teenagers by investigating the chain mediating roles of information retrieval and social interaction, aiming to identify optimal ways for adolescents to benefit from digital learning.

**Methods:**

A chain mediating effect were analyzed using a Hong Kong sample taken from the Programme for International Student Assessment (PISA) 2022 data pertaining to 4,378 15-year-old students from 163 schools.

**Results:**

The results indicated that (1) ICT use was positively correlated with SRL (β = 0.28, *p* < 0.001), (2) information retrieval and social interaction played independent mediating roles in the relationship between ICT use and SRL (effect values: 0.035 and 0.006, respectively), and (3) there was a chain effect between ICT use and SRL mediated by information retrieval and social interaction, with a total effect value of 0.235.

**Discussion:**

The study contributed to the existing theoretical framework by empirically validating a chain mediation model that integrated cognitive and social dimensions, thereby offering a more comprehensive understanding of the mechanisms through which ICT use influenced SRL. These findings could provide insightful information to the implementation of educational decisions to administer ICT tools to facilitate students’ SRL process.

## 1 Introduction

Self-regulated learning (SRL) is a critical competence for living and working in our increasingly complex and unpredictable world (Morris and Rohs, 2023). With the rapid development of information and communication technologies (ICT) such as cloud computing, big data and artificial intelligence, more opportunities have been provided for learners to enhance their SRL process (Sumuer, 2018; Zhang and Hew, 2025). ICT facilitates SRL by providing diverse types of learning applications (Aminatun and Oktaviani, 2019; Aisyah et al., 2021), abundant digital-learning resources (Aminatun, 2019; Zhu, 2024), real-time contact with online experts and peers (Pimdee et al., 2023), and timely feedback on learning progress (Liu et al., 2022; Faza and Lestari, 2025). These features collectively enable students to identify their learning needs, select appropriate learning content, tools and resources, and evaluate their learning outcomes, which stimulate self-regulation in their learning process (Kitsantas and Dabbagh, 2011; Esiyok et al., 2024).

While some studies have also pointed out that the accessibility of learning resources contrasts sharply with the asymmetric development of students’ self-regulation abilities, with 43% of online learners falling into “digital distraction” due to a lack of metacognitive strategies (Barboza et al., 2016). Behind learners’ veneer of technological adeptness lies a concerning gap in self-management and metacognitive skills. According to Hyland and Kranzow (2011), students commonly suffer from goal-drifting and information overload in network environments. Moreover, excessive reliance on ICT may weak learners’ ability to think deeply. Lindebaum and Fleming (2024) indicated that GenAI appear to lessen individuals’ willingness and ability to engage in meaningful critical thinking about its output. Individuals dependent on digital tools often exhibit a tendency toward rote memorization rather than meaningful understanding, which restricts their SRL to a superficial level.

At present, the effects of ICT use on students’ SRL is still a debating point. Although some studies have confirmed a significant correlation between ICT use and students’ SRL ability (Cheung and Hew, 2015; Istifci and Goksel, 2022), this correlation was influenced by many factors (Xu and Zhu, 2023). For example, Zaki et al. (2024) indicated that students showed different levels of SRL under different types of information retrieval techniques. Similar conclusions could be found in researches of Rogers and Swan (2004) and Din and Haron (2018). While some studies have demonstrated that students skilled in using social media to create, organize, and share content showed higher self-directed learning ability (Dabbagh and Kitsantas, 2012; Dabbagh and Kitsantas, 2013; Dong et al., 2024). The complexity of mediating variables between ICT use and SRL was not yet clear (Ben-Eliyahu and Bernacki, 2015). This study attempted to examine the interrelations among ICT use, information retrieval, social interaction, and SRL. The research questions were as follows:

*RQ1*. What is the effect of ICT use on SRL?

*RQ2*. How does ICT use influence students’ SRL?

## 2 Literature review

### 2.1 The relationship between ICT use and SRL

SRL refers to one’s ability to plan and control one’s learning environment and learning process, which includes goal setting, self-monitoring, self-instruction, and self-reinforcement (Harris and Graham, 1999; Schraw et al., 2006; Schunk and Zimmerman, 1996). Studies indicated that students acted out more self-directed and self-managed behaviors when using ICT (de Sousa et al., 2012; Asfar and Zainuddin, 2015; Kute and Palsamkar, 2017; Samruayruen et al., 2013). ICT-assisted learning significantly improved students’ self-regulated skills (Bernacki et al., 2011; Schneckenberg et al., 2011; Rashid and Asghar, 2016; Onivehu et al., 2018; Sardi et al., 2025). Recent developments in artificial intelligence applications have shown promise in supporting learners’ self-regulation in online learning (Jin et al., 2023; Molenaar, 2022). According to Xu et al. (2024), ChatGPT could aid learners in cultivating non-cognitive skills, including self-determination and self-regulation, by providing tailored feedback. The digital learning environment created by ICT enabled students to accommodate their own needs in their own time, place, and pace, and consequently made it possible for them to have more control over their learning process (Douglass and Morris, 2014; Beach, 2017). They could choose the learning contents and learning methods based on their interests (Tullis and Benjamin, 2011; Snodin, 2013), and then actively participated in the learning program (Annetta et al., 2009; Chen et al., 2010; Junco et al., 2011). In sum, ICTs have the potential to provide flexible opportunities and capabilities for learners to facilitate SRL. Therefore, this study proposed a hypothesis as following:

*H1*: ICT use has a positive effect on SRL.

### 2.2 The intermediary role of information retrieval

Given the prevalent use of the Internet, students had convenient and persistent access to worldwide information resources for self-education. Technology-rich environments created a new need for learners to be knowledgeable about resource selection as well as the ability to manage the collection, management, and use of relevant information (Fahnoe and Mishra, 2013). Manzo and Umar (2024) indicated that students’ knowledge of ICT skills enabled them to enhance their ability to collect and use digital resources which inturn enabled them to engage in their self-learning. According to Canan Gungoren et al. (2019), information searching strategies are related with self-regulation, self-learning. Tseng et al. (2014) conducted a study to identify a relationship between online information searching strategies and self-regulative learning and found a significant positive correlation between these two variables. Therefore, the second hypothesis of this study was proposed as following:

*H2*: Information retrieval mediates the influence of ICT use on SRL.

### 2.3 The intermediary role of social interaction

Besides information retrieval, ICT played an important role in informal conversation, dialog, collaborative content creation, and knowledge sharing, which helped students be more autonomous in their own learning (McLoughlin and Lee, 2010). Callaghan and Bower (2012) found that social networking sites supported students’ SRL by enabling them to complete tasks autonomously with the opportunity to study collaboratively. By using ICT especially web 2.0 tools, students could explore, express, and share their understanding of content and self-reflect on their learning experiences independently and publicly (Kitsantas and Dabbagh, 2011; Robertson, 2011). What’s more, ICT made it possible for students to receive help, support, and feedback from experts and peers so as to guide their learning (Karakas and Manisaligil, 2012; Yang et al., 2025; Laka et al., 2025). Current digital technologies also allowed for newer ways of creating interacting spaces, allowing for greater flexibility to students in selection of approaches to learning, such as individually, collaboratively and so on (Fahnoe and Mishra, 2013). Clearly these had significant implications for the development of SRL. Consequently, the abilities of ICT had a significant potential to foster SRL through facilitating social interaction. Thus, the third hypothesis was produced:

*H3*: Social interaction mediates the influence of ICT use on SRL.

### 2.4 The chain intermediary role of information retrieval and social interaction

From the above analysis, students’ decisions on SRL practices might influence by the information they retrieved and their interaction with others. While further researches indicated that information retrieval was positively correlated with social interaction such as information sharing, exchange, and consultation (Savolainen, 2019; Robson and Robinson, 2013; Du, 2014). According to Yang et al. (2022), those who viewed themselves as capable to gather information were more likely to seek information, and this perceived ability was related to information sharing. Actually, information seeking was one of the earliest forms of SRL based on web1.0. As searches became more complex, two-way interaction and human guidance were also commonly involved (Mills et al., 2014). Information exchange and communications were affected by constrains inherent in individuals’ information literacy (Wilson, 1999). New media literacy skills of information seeking and sharing supported and enriched social interaction, for instance, collaborative interaction, task interaction. In the views of Ruthven (2008), not all people reacted to the same tasks in the same way for their differences in information searching. Hence, the fourth hypothesis was proposed as following:

*H4*: Information retrieval and social interaction play a chain mediating role in the relationship between ICT use and SRL.

**Figure 1:**
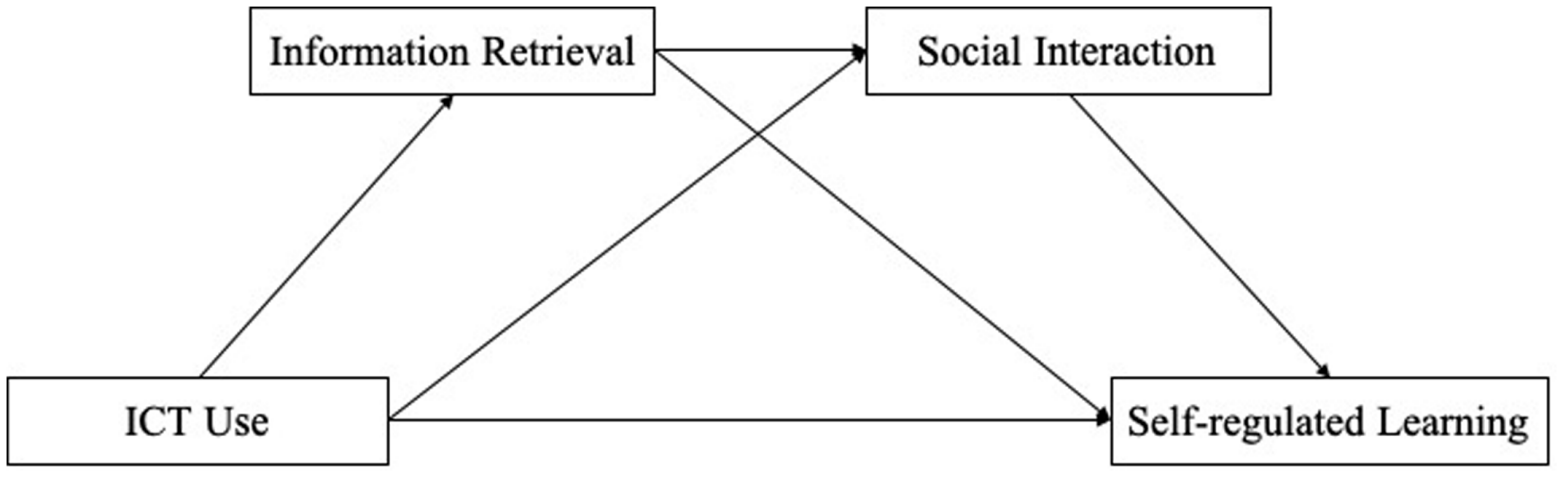
The chain intermediary model schematic.

Based on the analysis above, this study constructed a conceptual model as Figure 1.

## 3 Materials and methods

### 3.1 Participants

Analyses were conducted on data for Hong Kong derived from PISA 2022. A total of 163 schools were selected from Hong Kong in China. After removing data with missing values listwise, a total of 4,378 15-year-old students, who completed the student questionnaire and ICT familiarity questionnaire, were involved in this study. Among them, male students accounted for 51.70% (*N* = 2,262) and female students accounted for 48.30% (*N* = 2,116). The reasons for selecting this sample were as follows. First, this age group represented a critical developmental stage where SRL skills were increasingly essential for academic success and lifelong learning. Second, Hong Kong provided a unique educational context characterized by a high penetration of ICT infrastructure and a strong emphasis on digital literacy within its curriculum. Additionally, as a multicultural and technologically advanced region, Hong Kong offered valuable insights into how ICT use influenced SRL in environments where digital resources were widely accessible and integrated into daily educational practices. Since the variables of interest included four-, five-, and six- point Likert scales, this study unified all the variables into a five-point Likert scale by formula “Y = (B-A) * (x-a) / (b-a) + A” (IBM Support, 2024).

### 3.2 Variables

#### 3.2.1 ICT use

Four types of ICT use, including ICT use for school related activities during lessons, ICT use for school related activities outside classroom, ICT use for leisure activities during a typical week day, ICT use for leisure activities during a weekend day, were measured by ICT questionnaire in PISA 2022. Students were asked to indicate how often they used digital devices using a five- or six- point Likert scale. Standardized composite variables provided by the PISA 2022 dataset were used. The internal consistency reliability (Cronbach’s alpha) for 36 items was 0.93.

#### 3.2.2 Information retrieval and social interaction

Information retrieval refers to the process of finding information in such a way that non-relevant data were excluded while relevant information was found (Famaren and Tofi, 2021). While social interaction is defined as the process of exchanging information, ideas, emotions, or actions between individuals or groups in social setting (Azzaakiyyah, 2023). Students were asked to assess their ability to use ICT for information retrieval and social interaction using a five-point Likert scale from 1 (I do not know what this is) to 5 (I can easily do this) in ICT questionnaire in PISA 2022. For example, “search for and find relevant information online” was an item of information retrieval, while “share practical information with a group of students” was an item of social interaction. The internal consistency reliability (Cronbach’s alpha) was 0.84 and 0.92 for information retrieval and social interaction, respectively.

#### 3.2.3 SRL

SRL, also known as self-directed learning, was measured with a subset of items from student questionnaire in PISA 2022. By using a four-point Likert scale from 1 (not at all confident) to 4 (very confident), students were asked to indicate how confident they felt in self-planning, self-monitoring, and self-assessing their own learning process, such as “motivating myself to do school work,” “focusing on school work without reminders,” “assessing my progress with learning,” and so on. The reliability (Cronbach’s alpha) for this variable was 0.82.

#### 3.2.4 Covariates

Studies have indicated that students’ SRL has been influenced by their background information, such as gender (Stanikzai, 2020; Bezzina, 2010), social-economic background (Takabayashi, 2017). There was significant difference in SRL between students with different levels of economic, social, and cultural status (ESCS; Raimondi et al., 2025). Female students performed better in SRL than their male counterparts (Wijaya et al., 2020). Therefore, student’s gender and ESCS were considered as covariates and controlled in present study. Dummy variables were recoded for gender with 1 for girls and 0 for boys. In PISA 2022, the ESCS score was derived from three indicators: highest parental occupation status, highest parental education in years, and home possessions (Liu et al., 2024). And the ESCS score was reported after the transformation of having a mean of 0 and a standard deviation of 1 across senate-weighted OECD countries.

### 3.3 Statistical analytical procedure

SPSS 26.0 and its plug-in process were adopted to input, process, and statistically analyze the relevant data of this study. The data analysis was conducted through the following procedures. First, Descriptive and correlational statistics were used to examine the basic information about involve variables. Second, Harman’s single factor test was performed to detect the common method bias effect. Third, the main effect test was conducted based on the model 6 in the SPSS insert Process3.3 provided by Hayes (2013), with ICT use as the independent variable, SRL as the dependent variable, information retrieval and social interaction as the chain mediation variables, and sex and ESCS as the control variables. The whole regression equation was significant. Fourth, the bootstrap method was used to further test the mediating effect according to the (BootLLCI, BootULCI) judging whether the interval containing 0. If 0 is not included, the mediating effect is significant, while if 0 is included, it is not significant.

## 4 Results

### 4.1 Common method deviation analysis

Harman’s single factor test was used to exclude common method deviation caused by the questionnaire method (Zhou and Long, 2004). The results showed that there were 8 factors with eigenvalues greater than 1, and the variation explained by the first factor was 24.87% which was far below the critical value of 40% (Podsakoff et al., 2003), indicating that the effect of common method deviation would not influence the interpretation of data analysis results.

### 4.2 The descriptive statistics and correlation analysis between variables

To show the summary information of involved variables, the descriptive statistics and bivariate correlations among variables are presented in Table 1. Gender was positively associated with information retrieval and social interaction, indicating that girls had a better performance in information retrieval and social interaction than boys. ESCS was positively associated with ICT use, information retrieval, social interaction, and SRL, and the correlations ranged from 0.12 to 0.16. Therefore, gender and ESCS were considered as control variables in this study. ICT use was significantly positively correlated with SRL (*r* = 0.28, *p* < 0.001), which supported H1. At the same time, ICT use were positively correlated with information retrieval and social interaction (*r* = 0.30, *p* < 0.001; *r* = 0.31, *p* < 0.001). Information retrieval were positively correlated with social interaction and SRL (*r* = 0.83, *p* < 0.001; *r* = 0.30, *p* < 0.001). Social interaction was positively correlated with SRL (*r* = 0.29, *p* < 0.001).

**Table 1:** Descriptive statistics and correlations of variables involved in present study.

No.	Variables	*M*	SD	1	2	3	4	5
1	Gender	0.48	0.50					
2	ESCS	−0.41	0.99	0.02				
3	ICT use	2.74	0.61	0.01	0.15^***^			
4	Information retrieval	3.46	0.68	0.07^***^	0.13^***^	0.30^***^		
5	Social interaction	3.47	0.73	0.09^***^	0.12^***^	0.31^***^	0.83^***^	
6	Self-regulated learning	3.27	0.56	−0.03	0.16^***^	0.28^***^	0.30^***^	0.29^***^

^***^P < 0.001.

### 4.3 Analysis of the mediating effect

**Figure 2:**
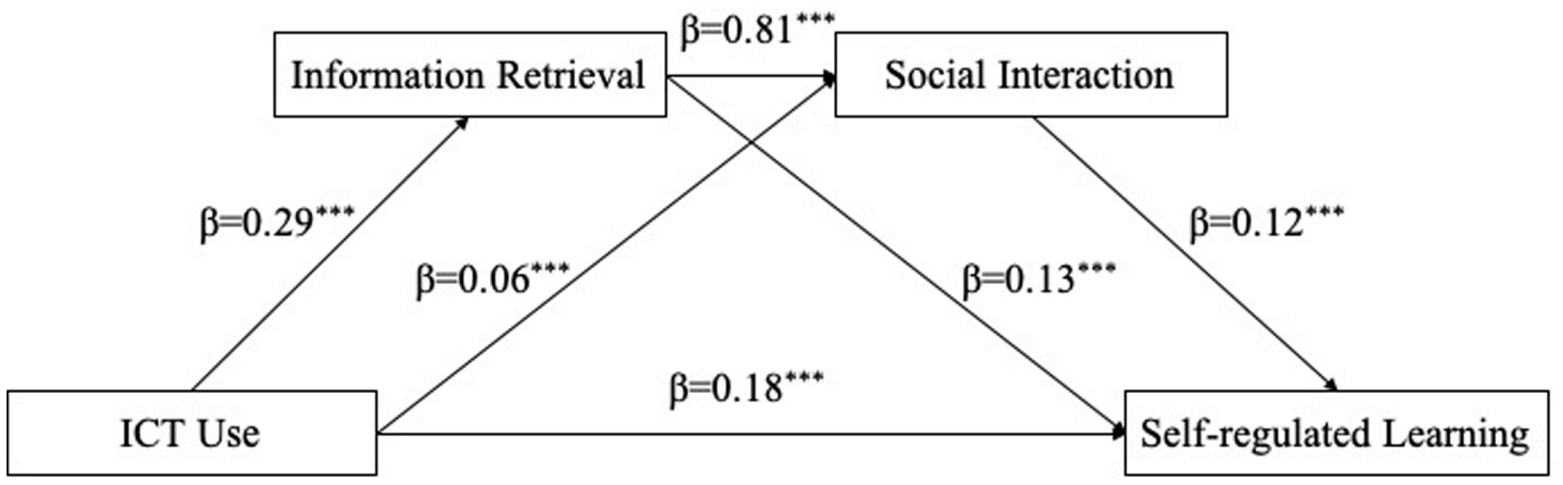
Chain mediation model of ICT use, information retrieval, social interaction, and self-regulated learning. ^***^p < 0.001.

According to the model 6 in the Process program developed by Hayes (2013), a chain mediation model was established with gender and ESCS as control variables, ICT use as an independent variable, information retrieval and social interaction as mediating variables, and SRL as a dependent variable (Figure 2). The overall regression analysis was significant, *R*^2^ = 0.09, *F*(4, 374) = 149.24, *p* < 0.001. As shown in the diagram, ICT use significantly and positively predicted information retrieval (β = 0.29, *p* < 0.001), and information retrieval significantly positively predicted SRL (β = 0.13, *p* < 0.001), indicating that information retrieval mediated the influence of ICT use on SRL. Therefore, hypothesis H2 was supported. At the same time, ICT use significantly and positively predicted social interaction (β = 0.06, *p* < 0.001), and social interaction significantly positively predicted SRL (β = 0.12, *p* < 0.001), which meant that social interaction mediated the influence of ICT use on SRL. Therefore, hypothesis H3 was supported.

By further testing the mediating effect, the bootstrapping method was used to re-sample 5,000 times to calculate for a 95% CI. As shown in Table 2, information retrieval and social interaction played an intermediary role between ICT use and SRL, and the total mediating effect was 0.235, while the 95% CI was [0.209, 0.262]. Three paths constituted this mediating effect. The first path, ICT use → information retrieval → SRL, showed an indirect effect value of 0.035 with a 95% CI of [0.021, 0.050]. This pathway underscored the cognitive mechanism through which technology supported SRL. Specifically, it suggested that strengthening students’ digital information literacy, such as the abilities to effectively search, evaluate, and organize online information, could enhance the beneficial influence of ICT on key SRL competencies such as planning, monitoring, and reflecting on one’s learning. The second path, ICT use → social interaction → SRL, demonstrated an indirect effect value of 0.006, with a 95% CI of [0.003, 0.010]. This pathway underscored the social dimension of learning with technology, implying that ICT tools which promoted collaborative discussions, peer feedback, and knowledge sharing could foster metacognitive and motivational aspects of SRL. The third path, ICT use → information retrieval → social interaction → SRL, yielded an indirect effect value of 0.026, with a 95% CI of [0.015, 0.037]. which demonstrated that information retrieval and social interaction played a chain mediating role in the relationship between ICT use and SRL. Therefore, hypothesis H4 was supported. This pathway revealed a sequential mechanism through which information retrieval enabled more meaningful social interactions, which in turn enhanced self-regulation. The findings indicated that educational interventions should not treat cognitive and social supports in isolation; rather, designing digital environments where students could first gather information and then engage in structured peer interactions or teacher-guided discussions was shown to create synergistic benefits for developing SRL competencies. Additionally, the results showed that the 95% CIs of the indirect effects differed significantly from zero, and that the mediating effects had statistical significance.

**Table 2:** Chain mediating effect on ICT use, information retrieval, social interaction, and self-regulated learning.

Model pathways		Effect value	BootSE	BootLLCI	BootULCI
Total effect		0.235	0.013	0.209	0.262
Direct effect		0.169	0.014	0.142	0.195
Indirect effect	IU → IR → SRL	0.035	0.008	0.021	0.050
	IU → SI → SRL	0.006	0.002	0.003	0.010
	IU → IR → SI → SRL	0.026	0.006	0.015	0.037

IU, ICT use; IR, Information retrieval; SI, Social interaction; SRL, self-regulated learning.

## 5 Discussion

### 5.1 The influence mechanism of ICT use on SRL

In this study, gender and ESCS were considered as covariates and controlled to test the effect of ICT use on SRL. It was found that ICT use was positively correlated with SRL, which is consistent with the previous research results (Popa and Topală, 2018) that the frequency of ICT use will affect SRL. With the professionalization of ICT use, students’ ICT literacy will also affect their SRL (Jannah, 2019). Istifci and Goksel (2022) addicted that students’ SRL perception levels increase in parallel with their ICT literacy skill levels. While few studies have been conducted to discuss the internal mediating mechanism of the impact of ICT use on SRL. This study explored the mediating role of information retrieval and social interaction in the relationship between ICT use and SRL. The results showed that information retrieval and social interaction formed a chain mediation pathway, partially explaining the overall association of ICT use and SRL.

### 5.2 Mediating effect of information retrieval

The results showed that information retrieval mediated the relationship between ICT use and SRL, suggesting that effective information retrieval skill is a crucial mechanism through which technology facilitates autonomous learning. According to Manzo and Umar (2024), students who actively engage with ICT tools demonstrate greater proficiency in accessing digital and electronic resources. The higher ICT use level means that they are more familiar with the format and methods of the medium used to present or make the resources available, which means they are more likely to develop proficient information retrieval strategies (Badilla Quintana et al., 2012). While, the improvement of the information retrieval level plays a pivotal role in determining whether learners can successfully regulate their learning processes (Tseng et al., 2014). According to social cognitive theory of self-regulation (Bandura, 1991), learners’ ability to access information efficiently strengthens their self-efficacy, which is the key driver of SRL. Therefore, information retrieval serves as a vital bridge connecting ICT use with SRL.

### 5.3 Mediating effect of social interaction

The research results demonstrated that social interaction mediated the relationship between ICT use and SRL, revealing a critical psychosocial pathway by which digital technologies enhance autonomous learning. First, ICT facilitates students’ access to expert guidance, peer support, and formative feedback, thereby fostering more effective SRL processes (Karakas and Manisaligil, 2012). Studies confirm that students who participate in structured digital discussions (e.g., forum-based peer reviews) develop stronger self-monitoring skills and task persistence (Balaji and Chakrabarti, 2010; Ickes et al., 2006). Second, ICT-enabled social interactions provide scaffolding for SRL strategies. For instance, real-time collaboration tools allow students to co-construct knowledge while observing peers’ problem-solving approaches, thereby strengthening the connections between knowledge construction and metacognitive regulation (Ouyang et al., 2021). Therefore, high levels of social interaction serve as a critical mechanism for fostering SRL skills through ICT use.

### 5.4 The chain-mediating effect of information retrieval and social interaction

The results of the current study revealed that ICT use had an impact on SRL through the chain mediation of information retrieval and social interaction. First, proficient information retrieval skills developed through active ICT engagement enable students to efficiently access and evaluate digital resources, thereby establishing a foundation for metacognitive regulation (Crystal and Foote, 2011; Bowler, 2010). Empirical studies confirmed that students skilled in information retrieval tend to exhibited stronger metacognitive skills to plan, monitor, and adapt their learning processes (Reisoğlu et al., 2020; Karaoğlan Yilmaz, 2016). Second, these enhanced information retrieval abilities facilitated more meaningful social interactions in digital learning environments. For instance, students who can quickly locate relevant resources are better equipped to participate in online discussions, engage in peer feedback, and collaboratively solve problems (Tan and Goh, 2015; Lin and Tsai, 2011; Bellhäuser et al., 2022). Such interactions promote SRL by enabling observational learning and collective reflection (Torres et al., 2024). Notably, the chain mediation effect addresses a gap in prior literature. While earlier studies established independent links between ICT use and SRL (Asfar and Zainuddin, 2015; Kute and Palsamkar, 2017), or between social interaction and SRL (Findyartini et al., 2024; Yen et al., 2022; Alvi and Gillies, 2015), this study reveals how information retrieval influences social engagement, which then enhances SRL.

However, the mediation pathway is context-dependent. Poorly structured ICT environments (e.g., information-overloaded platforms or isolated learning tasks) may disrupt this chain. Interventions such as embedded retrieval scaffolds (e.g., AI-assisted search prompts) and structured collaboration protocols (e.g., role-based peer reviews) could strengthen these linkages (Ng et al., 2024). Future research should explore how individual differences and cultural factors moderate this mediation effect.

## 6 Practical implications and limitations

The findings of this study highlight the critical role of ICT use in fostering SRL through the mediating effects of information retrieval and social interaction. This expands existing research by revealing how technology supports both information acquisition and social engagement, thereby enhances students’ ability to regulate their own learning processes. Practical implications for educators and policymakers can be drawn from these results.

First, educational institutions should prioritize the integration of ICT tools that support both independent information retrieval and peer interaction. Platforms such as digital libraries, virtual laboratories, and collaborative learning software (e.g., discussion forums, shared document editing) should be made readily accessible to students. Training programs should also be implemented to help learners develop effective strategies for locating, evaluating, and synthesizing information, as well as engaging in meaningful online discussions.

Second, instructors should design learning activities that encourage students to utilize ICT for SRL. For example, blended learning models that combine online searching tasks with group-based problem-solving exercises can strengthen SRL skills by reinforcing the interplay between information acquisition and knowledge co-construction (Raes et al., 2016). Additionally, fostering a digitally enriched learning community, where students actively seek feedback, share resources, and reflect on their learning strategies, can further enhance self-regulation (Yen et al., 2022).

Finally, while ICT offers significant benefits for SRL, educators should be mindful of potential distractions and cognitive overload associated with digital learning environments. High-frequency of ICT use is often accompanied by multi-task operation, which challenges students’ distribution of cognitive resource and If mishandled, might result in anxiety (Lepp et al., 2014; Gorjón and Osés, 2023). Excessive use of ICT in learning is not an optimal choice, and significant cognitive gains could be achieved by using the complementarity between traditional learning techniques with ICT-based learning in different blended settings (Bhutoria and Aljabri, 2022). Therefore, Structured guidance on time management, goal setting, and selective use of technology should be incorporated into curricula to help students maximize the advantages of ICT while minimizing its drawbacks.

Several limitations of this study should be acknowledged. First, this study used a cross-sectional research design to explore the mechanism of ICT use on SRL. Cross-sectional research can explain causal relationships between variables, in part, but future studies should adopt longitudinal or experimental designs to further examine how these variables interact over time. Second, the reliance on self-reported data may introduce response biases, such as social desirability or inaccurate self-assessment of ICT use patterns. Future studies could incorporate behavioral data (e.g., learning analytics, log files from digital platforms) to provide more objective measures of students’ ICT engagement and its impact on SRL. Third, while this study focused on information retrieval and social interaction as key mediators, other factors such as motivation, metacognition, or cultural differences in ICT use may also influence SRL. Further research should explore additional mediators or moderators to develop a more comprehensive understanding of how ICT supports SRL.

## 7 Conclusion

The current study reveals a significant positive correlation between ICT use and SRL of teenagers. Information retrieval and social interaction play a significant mediating role between ICT use and SRL. There are three mediating paths: the separate mediating effect of information retrieval, the separate mediating effect of social interaction; and the chain mediating effect of information retrieval and social interaction. This study can provide insights for effectively improving the SRL skills of teenagers, and the results have an important positive role in encouraging teenagers to actively participate in ICT use.

## Data Availability

The datasets presented in this study can be found in online repositories. The names of the repository/repositories and accession number(s) can be found in the article/supplementary material.
